# Identifying category representations for complex stimuli using discrete Markov chain Monte Carlo with people

**DOI:** 10.3758/s13428-019-01201-9

**Published:** 2019-02-13

**Authors:** Anne S. Hsu, Jay B. Martin, Adam N. Sanborn, Thomas L. Griffiths

**Affiliations:** 1 School of Electronic Engineering and Computer Science, Queen Mary University of London, London, UK; 2 Department of Psychology, New York University, New York, NY USA; 3 Department of Psychology, University of Warwick, Coventry, UK; 4 Department of Psychology, University of California, Berkeley, Berkeley, CA USA

**Keywords:** Category representation, Markov chain Monte Carlo, Words representations, Image databases

## Abstract

With the explosion of “big data,” digital repositories of texts and images are growing rapidly. These datasets present new opportunities for psychological research, but they require new methodologies before researchers can use these datasets to yield insights into human cognition. We present a new method that allows psychological researchers to take advantage of text and image databases: a procedure for measuring human categorical representations over large datasets of items, such as arbitrary words or pictures. We call this method *discrete Markov chain Monte Carlo with people* (d-MCMCP). We illustrate our method by evaluating the following categories over datasets: emotions as represented by facial images, moral concepts as represented by relevant words, and seasons as represented by images drawn from large online databases. Three experiments demonstrate that d-MCMCP is powerful and flexible enough to work with complex, naturalistic stimuli drawn from large online databases.

**Electronic supplementary material:**

The online version of this article (10.3758/s13428-019-01201-9) contains supplementary material, which is available to authorized users.

Big data is transforming society and offers a significant opportunity for psychology research: Large databases of text and images are potentially rich resources for understanding human cognition. However, the current usefulness of big data in psychology is limited, in part because of its size—constructing psychological tasks that engage with large datasets is a challenge. Labels for images and other kinds of data can be collected through online experiments and crowdsourcing (Carvalho, Lease, & Yilmaz, 2011; Greengard, 2011; Howe, 2006; Kittur, Chi, & Suh, 2008; Kleemann, Vob, & Rieder, 2008), but there is still a need for methods that can extract the psychological content of large databases more efficiently and meaningfully. In particular, the relevance of a piece of data (e.g., text, image, or sound) to a given psychological concept is often more suitably represented as a subjective probabilistic judgment than as a binary label. Understanding the distributional representations of people’s mental categories is relevant both for automating tasks that simulate cognitive processes, such as for artificial intelligence applications and also for understanding principles of human psychology. For example, if one were building an emotion detector for automated conversational agents, one would need an estimate of how likely it would be that different combinations of features of spoken stimuli (combinations of words and tone) would be indicative of the category of anger. Another example is gaining insight into the biases that inform our social prejudices by understanding how likely various combination of profiling features are to be associated with the concept of “socially threatening,” and comparing this with actual statistics of criminal profiles. Capturing the labels corresponding to such subjective judgments becomes prohibitively time-consuming for large, discrete datasets. This article presents a new method for efficiently inferring the structure of psychological categories defined over arbitrarily large datasets, such as of text and images.

Taking the view that categories are often viewed as subjective probability distributions over stimuli (Ashby & Alfonso-Reese, 1995), categorical representations can be revealed through asking people for their judgments of how relevant a stimulus is to a category. Similarly, insight into categorical representations can be revealed through a set of stimuli in which each sample is labeled with a subjective probability rating that represents how relevant the sample is to the category. However, it is difficult to efficiently obtain such judgments over large sets of stimuli. One solution to this problem comes from the key insight that if an experimenter can draw samples from this subjective distribution, the samples will be concentrated on the relevant stimuli, and irrelevant stimuli will be ignored. Using this insight, Sanborn, Griffiths, and Shiffrin (2010) adapted the well-known Markov chain Monte Carlo (MCMC) algorithm from computer science to sample from subjective probability distributions, a method they called *Markov chain Monte Carlo with people* (MCMCP). MCMCP has been used to estimate the structure of categories defined on continuous, easily parameterized stimuli, such as stick-figure animals and basic fruit shapes (Sanborn et al., 2010) or computer-generated faces (Martin, Griffiths, & Sanborn, 2012; McDuff, 2010).

Although the introduction of MCMCP has made it easier to explore complex, high-dimensional representations, it requires parameterized stimuli, and thus is inappropriate for databases of discrete items. To allow exploration of representations over large sets of discrete items we introduce a new method called *discrete Markov chain Monte Carlo with people* (d-MCMCP). This method allows estimation of subjective probability distributions over arbitrary datasets, such as images or text snippets. The resulting distributions can be used to identify the structure of people’s psychological representations in these domains.

The outline of this article is as follows. The next section introduces the key ideas behind MCMCP. We then present our new method, d-MCMCP, for discrete sets of stimuli. The remainder of the article focuses on three experiments showing a range of applications of d-MCMP. The first experiment explores categories of *happy* and *sad* faces using real photographic images, allowing us to compare against previous results using the MCMCP algorithm (Martin et al., 2012). The second experiment applies d-MCMCP to text, exploring the words perceived to be representative of the concept of morality for people with liberal and conservative political affiliations (Graham, Haidt, & Nosek, 2009). The third experiment demonstrates how d-MCMCP can be applied to large databases of images, by investigating how people represent seasons.

## Markov chain Monte Carlo with people

A standard approach to measuring human categories is to ask people to rate how likely a stimulus is to be a member of a given category. However, this approach has two serious limitations. First, when datasets become too large, collecting individual ratings is impractical. Second, questions such as “How good an example is this of a *happy face*?” can be difficult to answer. A better-defined question is to ask for pairwise judgments—for example, “Which is a better example of a *happy face*?,” but collecting all possible pairwise judgments requires even more trials, on the order of *n*^2^ for *n* items.

To address these issues, we introduced a class of algorithms from computer science and statistics called MCMC. Although algorithms exist for sampling from certain distributions with well-defined mathematical forms—for example, a normal or binomial distribution—it is often difficult to directly generate samples from arbitrary probability distributions, especially distributions that are not readily described with a mathematical formula, such as subjective category representations over arbitrary stimuli. MCMC algorithms solve the problem of generating samples from arbitrarily complex probability distributions using a statistical method. These algorithms work by drawing samples from a simpler distribution (from which direct samples are possible; e.g., a Gaussian or binomial distribution), and keeping or discarding these samples on the basis of a function of the difference between the simpler distribution and the target distribution. The samples that are kept result in a chain, and once the number of sequences in the chain is long enough, the frequencies represented in the chain become equivalent to those of samples from the true underlying distribution, *p*(*x*) (Gilks, Richardson, & Spiegelhalter, 1996).

A popular method for constructing Markov chains is the Metropolis–Hastings algorithm (Metropolis, Rosenbluth, Rosenbluth, & Teller, 2012). The sequence of the chain’s state is initialized at an arbitrary value, *x*. The next value in the sequence is generated via a two-step process. First, a candidate for the next value, *x'*, is chosen by sampling from a proposal distribution conditioned on *x*, *q*(*x'*; *x*)—for example, a normal distribution with mean *x*. Second, a decision is made as to whether that proposed value will be accepted, using an acceptance function that evaluates the relative probability of *x* to *x'* under the target distribution *p*(*x*). For example, one might compare the value of *p*(*x*) to the value of a normal distribution with mean *x* evaluated at *x'*. If the proposal is accepted, the state of the chain moves to *x'*, or else it remains at *x*. This process continues until the chain converges to its stationary distribution.

A variant of the Metropolis–Hastings acceptance function is at the heart of MCMCP. In a standard categorization task, people make a series of pairwise decisions, choosing the best category member from two proposed stimuli. The stimuli in each pair actually correspond to the values *x* and *x'* in the Metropolis–Hastings algorithm. The choices that people make determine which proposals are accepted. If we offer people a choice between *x* and *x'* and they choose *x'* with probability

2 $$ {P}_{choice}\left({x}^{\prime };x|c\right)=\frac{p\left({x}^{\prime }|c\right)}{p\left({x}^{\prime }|c\right)+p\left(x|c\right)}, $$

where *p*(*x* | *c*) indicates the degree to which stimulus *x* is perceived to be a member of category *c*, then their judgment is equivalent to the Barker acceptance function (Barker, 1965). A Markov chain based on this acceptance function will converge to *p*(*x* | *c*). Fortunately, Eq. 2 is a well-known model of human choice probabilities, referred to as the *Luce choice rule* or *ratio rule* (Luce, 1963; Shepard, 1987). This rule has provided a good fit to human data for people choosing between two stimuli on the basis of a particular property (Bradley, 1954; Clarke, 1957; Hopkins, 1954), and is also the decision rule used in many models of cognition (Ashby, 1992; McClelland & Elman, 1986; Nosofsky, 1986, 1987). With enough of these decisions, MCMCP converges to the probability distribution associated with that category, and individual stimuli will be encountered with probability *p*(*x* | *c*). On the basis of this correspondence, the MCMCP method implements Metropolis–Hastings by using people’s choices to determine which proposals are accepted (Sanborn et al., 2010). Furthermore, a variety of different assumptions for how participants are responding in proportion to Bayesian probabilities result in the same decision rule. For example, motivated participants can produce behavior that is more deterministic than probability matching. This kind of behavior is usually modeled using an exponentiated Luce choice rule, in which each term on the right-hand side of Eq. 2 is raised to an exponent *γ* (Ashby & Maddox, 1993). Previous work (Sanborn et al., 2010) has shown that if choices are made according to the exponentiated Luce choice rule, then MCMCP (and, so, also d-MCMCP) will converge to the distribution *p*(*x*)^*γ*^. Thus, if *γ* is unknown, we can only estimate *p*(*x*) up to a constant exponent, meaning that the relative probabilities cannot be directly evaluated, but the probabilities of different objects will remain in the same order. The efficiency of the d-MCMCP algorithm will depend on the target distribution, and thus on *γ* as well.

Although MCMCP is able to tackle large datasets by asking people well-defined questions, it introduces a new issue: MCMCP requires a symmetric proposal distribution *q*(*x'*; *x*), meaning that the probability of choosing a proposal given the current state would be the same if the proposal and the current state were reversed. When stimuli are described by a fixed set of parameters, this is easy—Gaussian or uniform distributions can be used to generate proposals for continuous parameters, and multinomial distributions can be used for discrete features. However, datasets consisting of images and text are not composed of easily parameterized items. In the remainder of this section we present a scheme for making reasonable proposals for exploring stimuli that are not easily parameterized. We call this method d-MCMCP.

The d-MCMCP method requires three additional steps. The first step is obtaining a database of stimuli. The second step is computing a symmetric similarity matrix, *S*, as a rough measure of the similarity between all possible item pairs. The similarity metric is chosen as is appropriate for the domain and need only provide a rough guide to the perceived similarity of human participants. For example, similarity between color histograms can be used to quantify the similarity of color images. The third step is constructing a graph of the stimuli based on these similarities. The d-MCMCP proposal distribution is then defined by a random walk on this graph. How well these assumed similarities correspond to the similarities of the human participants does not affect the algorithm in the long run: As long as the resulting graph is fully connected, free of closed cycles, and aperiodic, we are guaranteed to converge to the stationary distribution over time. Only the quality of the match between the graph and people should affect the algorithm’s efficiency, and indeed the efficiency of d-MCMCP does degrade as the difference between the assumed and actual similarities becomes large. See the supplementary materials for simulation results relating to the effects of noise in the similarity measure used.

To produce symmetric proposals using a random walk on a graph, the edges must be symmetric (i.e., the walk can traverse an edge in each direction), and each node in the graph must have the same degree (i.e., each node must have the same number of neighbors). Just choosing the *b* most similar neighbors for each node does not suffice, because although node *i* might be one of *b* nearest neighbors of node *j*, the reverse is not necessarily true, and the nodes could have different degrees.

To address this issue, we construct the graph that maximizes the similarity along edges while keeping the degree of each node constant. Formally, we want to find

$$ \arg {\max}_G{\sum}_{ij}{G}_{ij}{S}_{ij}\;\mathrm{such}\kern0.17em \mathrm{that}\;{\sum}_{ij}{G}_{ij}=b;{G}_{ii}=0;{G}_{ij}={G}_{ji}, $$

where *G* is the adjacency matrix of the graph, with *G*_*ij*_ = 1 if there is an edge from *i* to *j*, and *G*_*ij*_ = 0 otherwise. This is an instance of the *maximum-weight b-matching* problem (Papadimitriou & Steiglitz, 1998). Exact algorithms exist for solving this problem, such as the *blossom* algorithm (Edmonds, 1965), but these are impractical for large-scale applications. Consequently, we use an approximate algorithm based on max-product message-passing in order to find a *b*-matching (Jebara & Shchogolev, 2006).

**Fig. 1:**
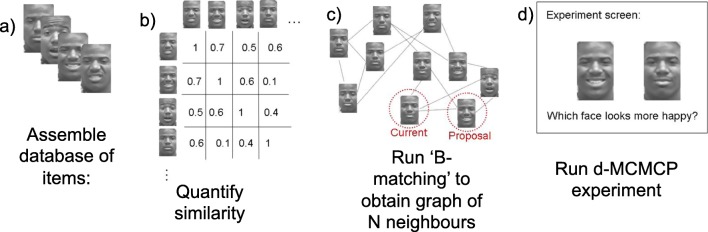
Steps for measuring category representations using d-MCMCP. (**a**) Assemble the database of items that are candidate category members. (**b**) Quantify similarity (using a suitably chosen metric) for all item pairs in the database. (**c**) Enter the similarity matrix into a *b*-matching algorithm to obtain a graph in which each item is connected to its *N* neighbors. (**d**) Run d-MCMCP using the neighbors as nearby proposals

Given this similarity graph, proposals for the d-MCMCP algorithm can be made in many ways. The most straightforward is to choose a proposal uniformly from all *b* neighbors, where the value of *b* is chosen at the experimenter’s discretion. Alternatively, an iterative geometric proposal of step length *n*_geom_ can be chose. Here, the proposal is generated iteratively using a number of steps, *n*_geom_, that is chosen from a geometric distribution with a fixed parameter. A random walk of length *n*_geom_ is then performed, choosing the next node uniformly from the *b* neighbors of the most recent one, and the proposal is the node at the end of the random walk. Geometric proposals allow a wider search over the stimulus space than with the uniform proposal. Additionally, it is prudent to allow for some small probability of choosing uniformly from all possible stimulus items, to allow the algorithm to move between local maxima. See Fig. 1.

D-MCMCP expands on the initial possibilities of the MCMCP method to measure distributional mental representations over real discrete datasets. As we discussed above, our representation of structure in the world is probabilistic, and thus the most complete understanding of our mental representation of world structures comes from a distributional representation. Furthermore, the distributions underlying many of our representations are complex and may be bimodal or have nonlinear interactions in the feature space. For example, an exceptionally quiet and an exceptionally loud voice could both be indications of upset emotions. As another example, larger containers may be preferred for cheaper olive oils, whereas smaller containers are preferred for high-end varieties. D-MCMCP offers the capability to efficiently reveal a richer and more accurate understanding of the distributions that underlie our categorical representations.

## Experiment 1: Happy and sad faces

As a first test of d-MCMCP, we examined the categories of *happy* and *sad* faces using images of real faces. Previous work had applied the MCMCP method to estimating these categories using parameterized face stimuli, where a continuous space was derived from eigenfaces computed from a set of images (Martin et al., 2012). We used the same image database to directly compare the results of d-MCMCP and MCMCP on a matched stimulus set. Example code and the face stimuli can be found at https://osf.io/u5sz4/.

### Method

#### Participants

A total of 60 undergraduates participated in exchange for course credit. Sample sizes were selected on the basis of previous experience with this experimental paradigm.

#### Stimuli

A database of 1,271 images of faces was assembled from the California Facial Expression (CAFE) database, a collection of 1,280 normalized 40 × 64 pixel grayscale portraits containing 64 individuals (Dailey, Cottrell, & Reilly, 2001). These images express approximately eight distinct “FACS-correct” emotions, classified according to the taxonomy of the Facial Action Coding System (Ekman & Friesen, 1978). Details of how the graph was constructed are available in the supplemental online material.

#### Procedure

Face images were convolved with Gabor filters at eight scales and five orientations. Principal component analysis (PCA) was then applied to the whole set of convolved images, and the Euclidean distance between the top 50 components was used as the similarity metric for defining the matrix *S*. Two graphs *G* were produced using the approximate *b*-matching algorithm from Jebara and Shchogolev (2006), one with *b* = 6 and one with *b* = 16. This algorithm gives an approximate solution to the *b*-matching problem, so there was still some minor variation in the degree of individual nodes. Our empirical evaluation of the performance of the d-MCMCP procedure will thus also help indicate whether this residual variation affects the results. There is no guarantee that a maximal *b*-matching will be connected, so we used the largest connected component as the basis for the d-MCMCP procedure. The largest connected components contained 1,216 images with *b* = 6, and all 1,271 images with *b* = 16.

We compared three different methods for defining the proposal distributions, run as three separate conditions. For all three proposal methods, we allowed a 10% chance of proposing a jump to a node chosen uniformly at random. The three methods for choosing the remaining proposals were uniform random walk on the graph with *b* = 6 (U6), uniform random walk on the graph with *b* = 16 (U16), and the geometric proposal with *n*_geom_ = .5 on the graph with *b* = 6 (G6).

Participants were randomly assigned to conditions. Trials were presented on three different computers, one for each proposal type. Following previous work, each participant completed trials corresponding to different d-MCMCP chains (Martin et al., 2012; Ramlee, Sanborn, & Tang, 2017). There were four chains: two for *happy* faces and two for *sad* faces. There were 100 trials for each of the four chains. On a given trial, the participant decided which of a pair of faces was either more *happy* or more *sad*. Twelve initial practice trials were not included in the analysis. Forty catch trials were presented of face pairs for which the more *happy* or *sad* face was clearly obvious (in this case, we used the emotion designations in the CAFE database to select faces that should be clearly happy or sad). Thus, each participant responded to 100 × 4 + 12 + 40 = 452 trials, which took approximately 25 min. The responses were linked in chains of ten participants each: The last trial of each of the four chains was passed along to the next participant as his or her first nonpractice trial, to form a linked chain of 1,000 trials. Participants who did not correctly answer at least 27 of the catch trials (*p* < .01 under random guessing) were not included in the results or added into a chain. We collected two chains of ten participants for each proposal type, corresponding to four *happy* and *sad* chains with 1,000 trials in each chain.

### Results

**Fig. 2:**
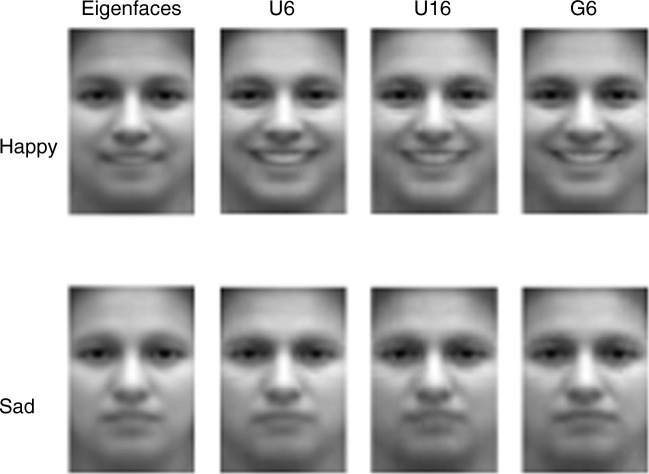
Average faces for MCMCP (eigenfaces) and for each type of d-MCMCP proposal. Averages are taken across all trials and all four chains corresponding to *happy* and *sad*

The previously used MCMCP method drew samples generated from a continuous eigenface space, and thus did not result in ratings being applied directly to the discrete images from the dataset. Previous work showed by averaging the generated images in the chain that the samples generated by MCMCP more quickly converged to being representative of people’s mental representations of each category than did alternative methods (i.e., reverse correlation; Martin et al., 2012). Here, to directly compare the previous MCMCP with the present d-MCMCP results, the images selected on each d-MCMCP trial were averaged together to produce the average faces shown in Fig. 2. All three proposal methods produced mean faces that appeared reasonably consistent with the target emotions. Also included in Fig. 2 are the results reported in Martin et al. (2012), using MCMCP in a parameterized space based on the eigenfaces derived from the image database we used for d-MCMCP. Qualitatively, the results from d-MCMCP are at least as good as, and perhaps better than, those produced using eigenfaces.

To quantify the performance of the different variants of the algorithm, we conducted a follow-up experiment in which a group of 40 participants recruited via Amazon Mechanical Turk provided ratings of the emotions exhibited by the faces derived from our chains. For each proposal type (and for the chains based on eigenfaces used in Martin et al., 2012), cumulative average faces were computed for each of 40 logarithmically spaced numbers of trials, averaging across all four chains that corresponded to each emotion. Trial numbers greater than 50 images were averaged only over the 50 most recent trials, meaning that no more than 200 faces contributed to any single image. Participants rated the emotion exhibited by each of these mean faces on a scale from 1 to 9, where 1 indicates *very sad* and 9 indicates *very happy*. All participants rated all faces and received $1 in compensation for their time. No participant data were excluded.

**Fig. 3:**
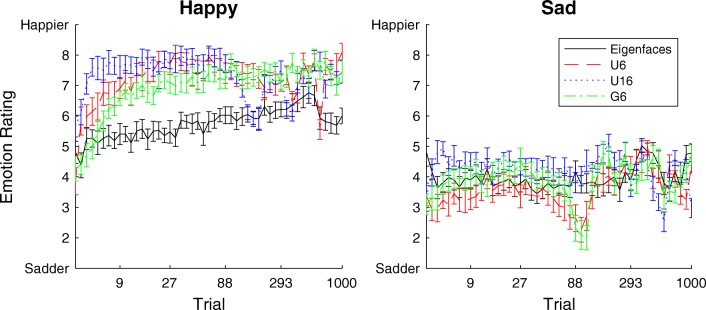
Comparison of MCMCP and d-MCMCP: MCMCP using eigenfaces compared with d-MCMCP with a variety of proposal methods, executed on the same set of face stimuli. Happiness ratings are shown for average faces for the three types of d-MCMCP proposals as well as for the original MCMCP method as a function of trial number (error bars show 95% confidence intervals). Averages are taken across the 50 most recent trials (or starting from the first trial, for trials less than 50) and across all four chains corresponding to the same emotion, *happy* or *sad*. Also included are face ratings for the results of a previous MCMCP experiment that used eigenfaces derived from the same image database (Martin et al., 2012).

The results of our follow-up experiment are shown in Fig. 3. The d-MCMCP method results in statistically significantly higher ratings for faces derived from *happy* chains, regardless of the proposal type, perhaps as a consequence of being able to explore a larger space of faces than the eigenface method. Looking at the 95% confidence intervals of differences in the mean ratings across all trials (i.e., each participant’s ratings across all trials are averaged into one rating), a d-MCMCP face is significantly more likely to be perceived as happy for all proposal types (positive values are “better” ratings for happy faces): *CI* = [1.18, 1.68], *d* = 1.43 for U6; *CI* = [1.36, 1.82], *d* = 1.59 for U16; *CI* = [0.95, 1.40], *d* = 1.17 for G6. The results for *sad* chains are more comparable to the eigenface method (negative values are “better” ratings for sad faces): U6 was significantly better, *CI* = [– 0.64, – 0.11], *d* = *–* 0.37; U16 was significantly worse, *CI* = [ 0.023, 0.52], *d* = 0.27 ; and G6 was not significantly different, *CI* = [– 0.35, 0.17], *d* = *–* 0.092. For both *happy* and *sad* chains, there is some variation in the emotion ratings of mean faces over time, consistent with MCMCP exploring the distribution of faces associated with the category (and possibly moving between modes of that distribution) rather than finding the most extreme instance of that category.

## Experiment 2: Moral foundations

Our second experiment applied d-MCMCP to concepts in text. Here, we used d-MCMCP to examine and further support a theory from social psychology that conservatives and liberals have different concepts of morality. In particular, conservatives’ moral intuitions have been claimed to reflect values in the five “moral foundations” of harm/care, fairness/reciprocity, ingroup/loyalty, authority/respect, and purity/sanctity, whereas liberals primarily endorse only the first two principles (Graham et al., 2009). This difference has previously been observed in abstract assessments of moral judgments of statements and scenarios, reactions to taboo trade-offs, and use of foundation-related words in the moral texts of sermons in liberal versus conservative churches. Here, we examined differences in the concept of morality between liberals and conservatives by employing d-MCMCP over a set of words.

### Method

#### Participants

A total of 251 US-based participants were recruited via Amazon Mechanical Turk and were paid $1 each for their time. The sample sizes were selected on the basis of previous experience with this experimental paradigm.

#### Stimuli

In Graham et al. (2009), a dictionary of words was assembled that were closely related to each of the moral foundations. We chose 32 of these words from each of the five foundations, for a total of 160 words.

#### Procedure

Similarities between all possible word pairs were measured using latent semantic analysis results made available online at http://lsa.colorado.edu/cgi-bin/LSA-matrix.html (Landauer & Dumais, 1997). The similarity between all pairs was represented as a similarity matrix that was fed into the *b*-matching algorithm. A graph was found using *b* = 10, which was roughly between the values used in Experiment 1. We used a proposal distribution corresponding to a uniform random walk on this graph. Also, as in Experiment 1, we allowed for a 10% chance of proposing a jump to a node chosen uniformly at random. Each participant completed trials corresponding to four d-MCMCP chains, all of which explored the single concept of “morality.” On a given trial, participants were given the choice of two words and answered the question: “Which of these two words is most relevant to morality?” We used a proposal distribution corresponding to a uniform random walk on this graph, but with a 10% chance of proposing a jump to a node chosen uniformly at random. There were 100 trials for each of the four chains. Twelve trials in the beginning were offered as practice, which were not included in the analysis. Thus, each participant responded to 100 × 4 + 12 = 412 trials, which took approximately 25 min. The responses were not linked between participants (as had been done in Exp. 1). At the end of the experiment, following Graham et al. (2009), participants were also asked “Which best describes your political identification?” and given the choices (1) *extremely liberal*, (2) *somewhat liberal*, (3) *middle*, (4) *somewhat conservative*, (5) *extremely conservative*, and (6) *none of these*.

### Results

**Fig. 4:**
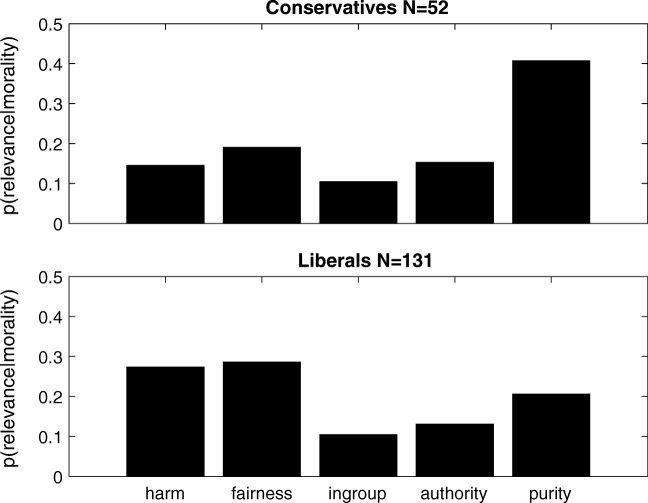
Relevance of moral foundations for conservatives versus liberals: Results of d-MCMCP over words that are associated with the five moral foundations hypothesized by Graham et al. (2009) for participants who self-identified as conservatives (top) versus liberals (bottom). The values *p*(relevance | morality) are normalized counts—that is, the proportions of times words associated with the given moral foundation were chosen as being more relevant to the concept of morality.

Because we were interested in contrasting responses between people who self-identified as conservatives versus liberals, we only analyzed the responses of participants who responded *extremely liberal* and *somewhat liberal* (classified as liberals, *N* = 131) or *somewhat conservative* or *extremely conservative* (classified as conservatives, *N* = 52) to the political identification question. For each of these participants, we calculated the frequency with which words corresponding to the five foundations were selected. Figure 4 shows the normalized frequencies for conservatives (top) and liberals (bottom). Normalized frequencies over all 160 words, along with the 20 most morally relevant words for conservatives and liberals, are shown in Supplementary Figs. 2 and 3. Under the statistically conservative assumption that each participant counted as a single sample, the difference between the normalized histograms from the two conditions was significant by a Pearson’s *χ*^2^ test [*χ*^2^(4) = 9.76, *p* < .05]. A two-way analysis of variance (ANOVA) over moral foundations and political identification showed a main effect of foundations [*F*(4, 905) = 24.24, *MSE* = .95, *p* < .00001, *η*_p_^2^ = .097], as well as an interaction [*F*(4, 905) = 15.87, *MSE* = .62, *p* < .0001, *η*_p_^2^ = .066], supporting the idea that conservatives and liberals find different amounts of relevance in the five moral foundations. An ANOVA over foundations for conservative participants showed a main effect of foundations [*F*(4, 255) = 17.41, *MSE* = .74, *p* < .00001, *η*_p_^2^ = .79], with the foundation of purity (*M* = .41, *SD* = .31) being significantly more relevant (with Bonferroni correction) than the other four foundations: harm (*M* = .15, *SD* = .15), *t*(51) = 4.67, *p* < .0001, *d* = 1.08, *CI* = [.15, .37]; fairness (*M* = .19, *SD* = .21), *t*(51) = 3.45, *p* = *.*0011, *d* = 0.82, *CI* = [.091, .34]; in-group *M* = .10, *SD* = .14), *t*(51) = 5.57, *p* < .0001, *d* = 1.27, *CI* = [.19, .41]; and authority (.15, *SD* = .18), *t*(51) = 4.60, *p* < .0001, *d* = 1.015, *CI* = [.14, .36]. An ANOVA over foundations for liberal participants also showed a main effect of foundations [*F*(4, 650) = 23.36, *MSE* = .88, *p* < .00001, *η*_p_^2^ = .87]. Post-hoc analysis shows the foundation of purity (*M* = .21, *SD* = .22) being more relevant (with Bonferroni-adjusted significance) than in-group (*M* = .10, *SD* = .12), *t*(130) = 4.36, *p* < .0001, *d* = .57, *CI* = [.055, .15], and authority (*M* = .13, *SD* = .13), *t*(130) = 3.15, *p* = *.*0020, *d* = 0.41, *CI* = [.03, .12]. There was also a trend (not significant after Bonferroni adjustment) toward purity being less relevant than harm (*M* = .27, *SD* = .24), *t*(130) = – 2.03, *p* = *.*045, *d* = *–* 0.30, *CI* = [– .13, – .0017], and fairness (*M* = .29, *SD* = .23), *t*(130) = – 2.43, *p* = *.*016, *d* = *–* 0.36, *CI* = [– .14, – .015]. Harm and fairness were both significantly more relevant than the other three foundations. The foundation of harm was significantly more relevant (with Bonferroni adjustment) than in-group, *t*(130) = 6.91, *p* < 1e-09, *d* = .91, *CI* = [.12, .22], and authority, *t*(130) = 5.30, *p* < e-06, *d* = .75, *CI* = [.090, .20]. Harm also had a trend (not significant with Bonferroni adjustment) toward being more relevant than purity, *t*(130) = 2.0, *p* = *.*045, *d* = 0.30, *CI* = [.0017, .13]. Similarly the foundation of fairness was significantly more relevant (with Bonferroni adjustment) than in-group, *t*(130) = 7.38, *p* < 1e-10, *d* = 1.0, *CI* = [.13, .23] and authority, *t*(130) = 6.44, *p* < 1e-08, *d* = .84, *CI* = [.11, .20]. Fairness also had a trend (not significant with Bonferroni adjustment) toward being more relevant than purity, *t*(130) = 2.43, *p* = *.*016404, *d* = 0.35613, *CI* = [.015, .14].

These results are consistent with those of Graham et al. (2009), who found that conservatives believed that all five moral foundations were relevant to morality, whereas liberals tended to mostly value the foundations of harm and fairness. However, the relative importance of purity as compared with all other foundations for conservatives, and in comparison with in-group and authority, was not seen in Graham et al., for whom the relative importance of various foundations varied between experiments, suggesting an interesting direction for future research.

## Experiment 3: Seasons

In our third experiment, we used d-MCMCP to explore categories defined on images from a large online database. Specifically, we explored the categories associated with the seasons spring, summer, autumn, and winter, using 4,000 images obtained from online image databases. By applying the d-MCMCP procedure to these stimuli, we could identify high-probability images and compute informative aggregate statistics for each category, such as determining the distribution of colors associated with each season.

### Method

#### Participants

A total of 90 participants were recruited using Amazon Mechanical Turk. Each participant was paid $1 for completing the 25-min experiment. The sample size was selected on the basis of previous experience with this experimental paradigm.

#### Stimuli

A set of 4,000 colored season-related images was assembled by searching for public-domain web images using the phrases “spring season,” “summer season,” “autumn season,” and “winter season” in Google Image Search and on Flickr.com. The top 500 results for searches on Google and Flickr for each season were downloaded. All images were resized so that the maximum dimension was 250 pixels, while preserving the original ratio of image height to width.

#### Procedure

The similarity between all possible image pairs (7,998,000 pairs for 4,000 images) was quantified using both the basic color histogram (BCH) descriptor (Griffin, 2006) and the scale-invariant feature transform (SIFT; Lowe, 1999). BCH classifies and counts pixels as belonging to one of 11 basic colors (black, white, gray, red, orange, yellow, green, blue, purple, pink, and brown). SIFT applies local filters to transform images into collections of local feature vectors that are invariant to scaling, rotation, and translation of the image. Similarity results over all pairs of images for both methods were normalized to have unit variance and then added together, thus yielding a similarity measure that combined the results of both BCH and SIFT. The similarity between all pairs was represented as a similarity matrix that was fed into the *b*-matching algorithm. A graph was found using *b* = 5, which was the smallest value such that all 4,000 images remained fully connected. We used a proposal distribution corresponding to a uniform random walk on this graph.

Each participant made pairwise choices between images by answering questions such as “Which image is more representative of spring?” We used a proposal distribution corresponding to a uniform random walk on this graph. There were 100 trials for each of four chains, one for each season. There were also 12 practice trials, as well as 40 catch trials for which one image of the pair obviously corresponded to a particular season (as judged by the experimenter). Thus, each participant completed 452 trials. We included catch trials with the intention of excluding participants who did not get at least 27 of the catch trials correct (corresponding to a less than 1% chance that the answers were completely random guesses) from the chains and later analyses. All participants passed our criteria and were included in the analysis. We collected data by linking three sets of ten participants, forming three chains of 1,000 trials for each of the four seasons.

### Results

**Fig. 5:**
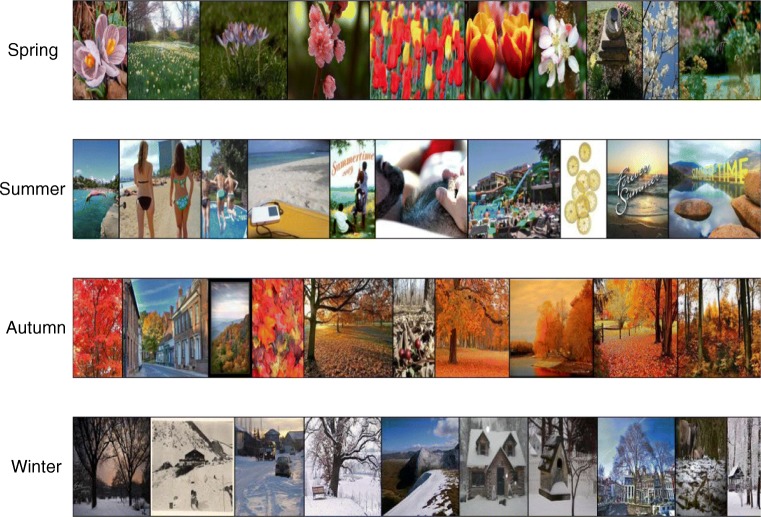
Seasonal images: The top ten most popular images over all chains for each season, decreasing in popularity from left to right.

**Fig. 6:**
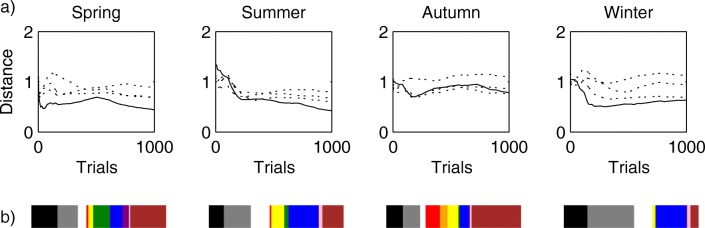
Color histograms for seasonal images. 11-bin color histograms were calculated for all cumulative images in all three chains as a function of the number of trials. (**a**) Average L1 distance between the cumulative histograms of a single chain and of the other two chains that correspond to the same season (solid line) or of another three chains that correspond to a different season (one dotted line for each other season). (**b**) Color histograms of all images, averaged over all chains for each season.

The top ten images that were chosen most often over all three chains for each season are shown in Fig. 5. Clearly, the images are very indicative of each season. Figure 6a shows, as a function of the number of trials, the L1 distance between 11-bin color histograms calculated for the cumulative images, both between chains for the same season and between chains corresponding to different seasons. The within-chain distance decreases over time and is typically lower than the similarity between chains, supporting the idea that chains are converging toward different parts of the space of images. Figure 6b shows a simple example of the kind of statistical analyses that can be done on the resulting samples. The color histograms for the different seasons are quite different from one another and correspond to palettes that intuitively match the seasons.

## Conclusion

We have introduced a new method that can be applied to arbitrary sets of items, making it possible to measure people’s representations of categories defined over those items. Of course, a wide variety of methods can be applied to eliciting categories from people. Our d-MCMCP method outperformed MCMCP on eliciting the categories of happy faces. Previous work has shown that MCMCP both outperforms complex regression and predicts an individual’s category preferences better than those of other individuals do (Huszár, Noppeney, & Lengyel, 2010). Thus, it seems likely that d-MCMCP would show similar advantage over these other approaches. In future work, it would be useful verify this by directly comparing d-MCMCP with these methods, as well as comparing it to approaches, such as active learning (Myung & Pitt, 2009) and cognitive tomography (Houlsby et al., 2013). D-MCMCP generally makes weaker assumptions about the decision-making process than do these other methods, so it may well prove more advantageous with real-world tasks and stimuli, a context in which the assumptions the other methods make about cognitive processes are less likely to be correct. Although our textual example used only words, one could easily extend the method to evaluate lines of texts, paragraphs, and documents. Furthermore, one can also use d-MCMCP to tag video clips and other data types. As with most data-driven models, the quality and generalizability of d-MCMCP’s results are influenced by biases present in the dataset used. For example, if one wanted to compare representative male versus female faces, and if the data had more examples of young female faces and of old male faces, the final categorical representations might be dominated by age-dependent rather than gender-dependent features. Thus, the limitations and biases present in the dataset used should be considered when making conclusions about mental representations. Our method is likely to be valuable to data scientists, as well as to machine-learning and computer vision researchers interested in training systems to automate categorizing images and other complex stimuli. Our method can be used to retrieve human judgments for concepts in a wide variety of domains, including stereotyped judgments from individual characteristics, consumer preferences as a function of variations in product offerings, or mood/conversational intent from word usage patterns. For psychologists, conducting experiments using d-MCMCP on a large scale will allow us to build up a catalog of human category representations, taking a step toward understanding how those categories are formed.

## Electronic supplementary material

ESM 1 (DOCX 3402 kb)
