# Supplementary material for: Identifying category representations for complex stimuli using discrete Markov chain Monte Carlo with people
Source: Behav Res Methods. 2019 Feb 13;51(4):1706–16. doi: 10.3758/s13428-019-01201-9 (PMC6691032; doi:10.3758/s13428-019-01201-9)
Supplement: Supplementary file 1 — (DOCX 3402 kb) [file 13428_2019_1201_MOESM1_ESM.docx]

Supplementary Materials:

**Making proposals**

Given a similarity graph, proposals for the d-MCMCP algorithm can be made in many ways. The most straightforward is to choose a proposal uniformly from all *b* neighbours, where the value of *b* is chosen at the experimenter's discretion. A second possibility is making a geometric proposal. Here, the proposal is generated iteratively using a number of steps, *n_geom_* that is chosen from a geometric distribution with a fixed parameter. A random walk of length *n_geom_* is then performed, choosing the next node uniformly from the *b* neighbors of the most recent one, and the proposal is the node at the end of the random walk. Additionally, it is prudent to allow for some small probability of choosing uniformly from all possible stimulus items to allow the algorithm to move between local maxima.

**Noise in similarity simulation**

To demonstrate the effect of noise in the similarity metric used to create the adjacency matrix, which would cause it to deviate from actual human perceived similarity, we ran a simulation. We first created a theoretical target distribution over 1024 hypothetical 2D stimuli whose features exist on a 32x32 grid. The target distribution is modelled as a 2D Gaussian distribution of SD_x = 1 and SD_y = 0.5 located at a mean value of x=10, y=20. We defined the pairwise similarity between stimuli S1 and S2 to be:

$$s\left( S_{1}, S_{2} \right)= e^{\sqrt{{{(X}_{1}-X_{2)}}^{2}+{{(Y}_{1}-Y_{2)}}^{2}}}$$

Where (X_1_, Y_1_) and (X_2_ Y_2_), were the X and Y values of S_1_ and S_2_ respectively. We used a proposal distribution that sampled uniformly from the 16 nearest neighbors. We ran simulations where noise was added to the pairwise similarity between all pairs of stimuli, and the simulated noise drawn from a random normal distribution with SD = .01, .1, .25, or .5. We ran 10 simulations for each level of noise, and in each simulation the MCMC chains ran for 50,000 trials each.

Supplementary Figure 1a shows the defined target distribution. Figure 1b shows average Euclidean squared distance from the target distribution as a function of the number of trials for each level of noise. For comparison we also show results with no noise and uniform random proposals. Our simulation results show that noise with SD = .01 or .1 did not make performance worse than no noise. However, with SD = .25, performance deteriorated towards that of random proposals, and with SD = .5 performance was actually worse than with random proposals. This is likely because with high SD the regions of high probability are scattered, and the graph structure means that the algorithm traversed between these high probability regions more slowly than it did with random proposals. Result for uniform proposals with 6 neighbors were comparable.


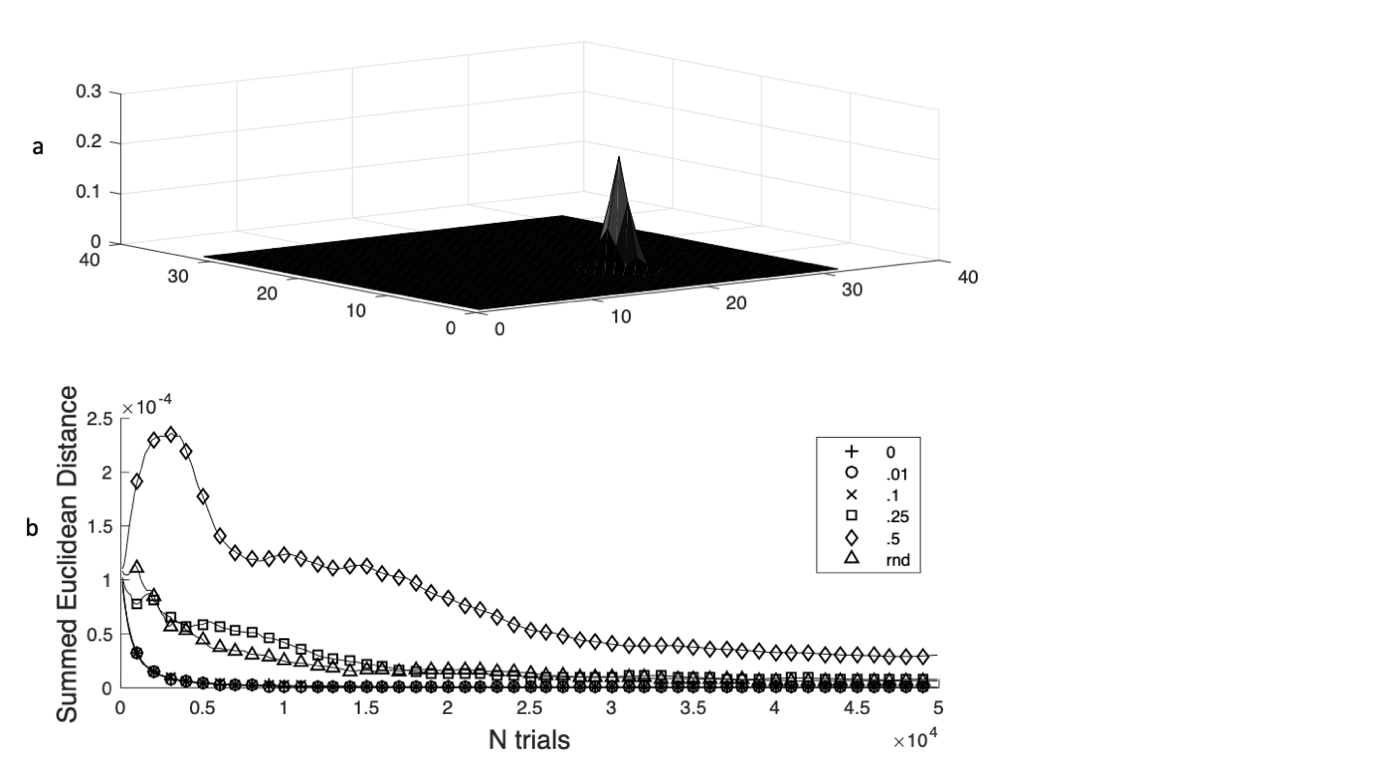


Supplementary Figure 1

a) Target distribution defined over 1024 stimuli whose features and probability values are shown in a 2D plot. b) Results of d-MCMCP simulations with 16 nearest neighbour uniform proposals where Gaussian Random noise is added to the similarity matrix with SD’s of 0.01, 0.1, 0.25, 0.5. Also shown are results with zero noise (0) and completely uniform random proposals (rnd).

**Morality results for individual words**


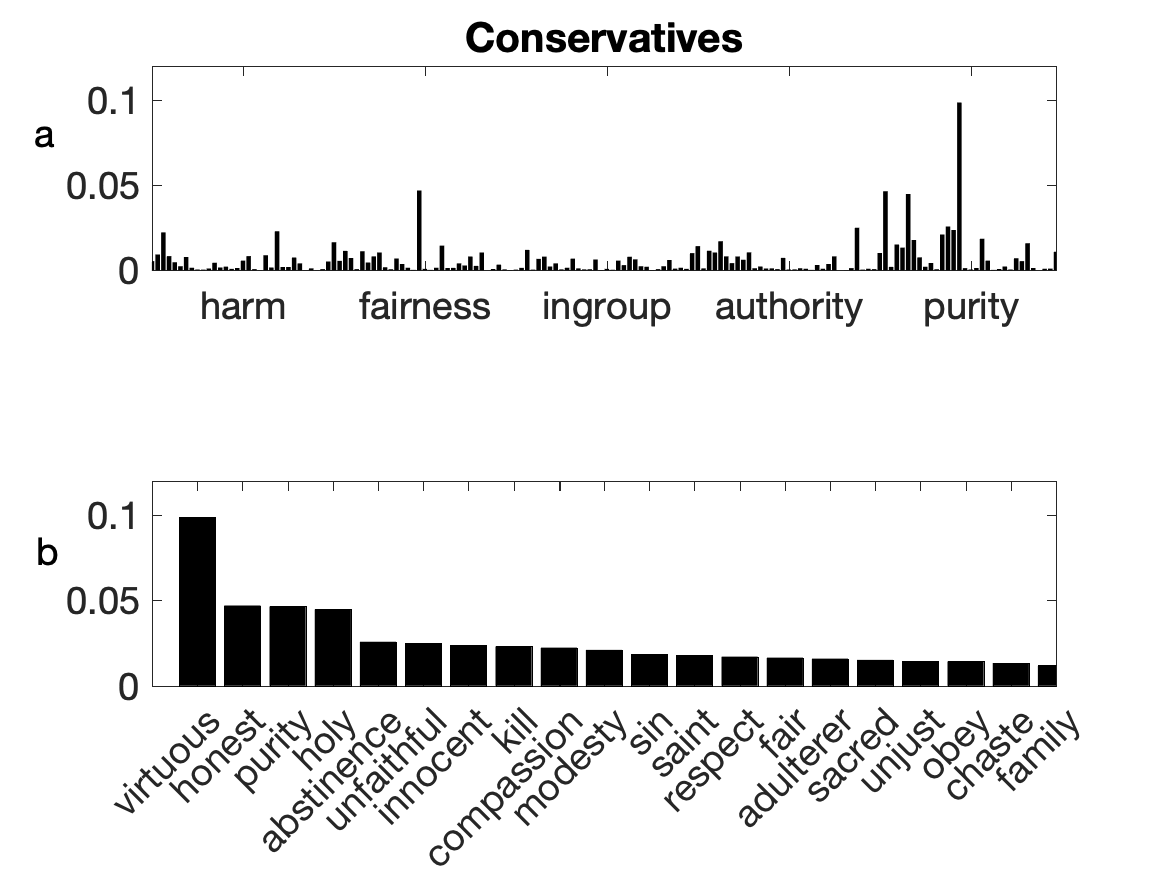


Supplementary Figure 2:

a) Distribution of samples for conservative participants over individual 160 words. There were 32 words chosen to represent each of the five moral foundations. B) Probabilities associated with the 20 most morally relevant words for conservative participants.


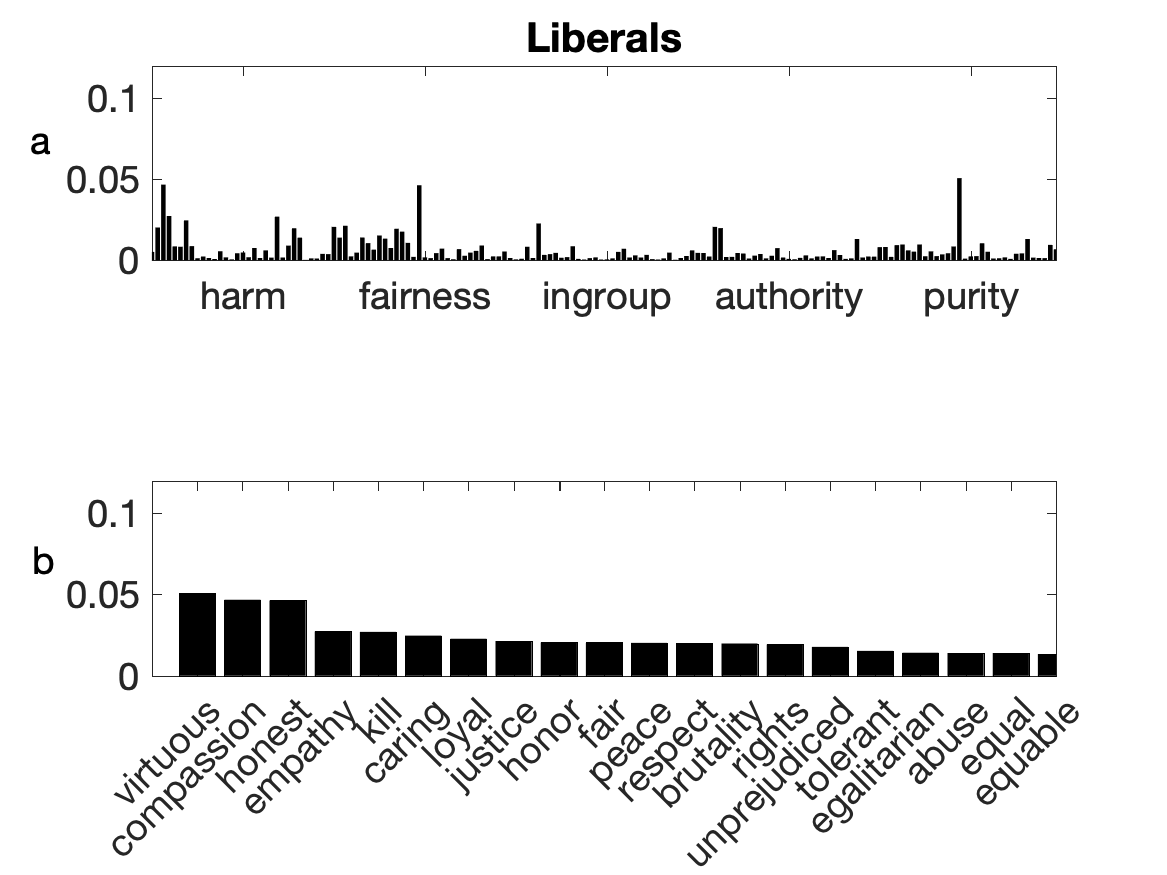


Supplementary Figure 3:

a) Distribution of samples for liberal participants over individual 160 words. There were 32 words chosen to represent each of the five moral foundations. B) Probabilities associated with the 20 most morally relevant words for liberal participants.
